# Preparation and Identification of Antioxidant Peptides in Quinoa

**DOI:** 10.3390/foods15173124

**Published:** 2026-09-02

**Authors:** Qian Zhang, Qian Du, Hui Li, Jing Yuan, Jie Zhao, Fengmei Sun

**Affiliations:** 1 College of Agriculture, Forestry and Technology, Hebei North University, Zhangjiakou 075000, China; qian203521@163.com (Q.Z.); 19511133103@163.com (Q.D.); lihui924@126.com (H.L.); 15931327017@163.com (J.Y.); 2 Hebei Key Laboratory of Quality & Safety Analysis-Testing for Agro-Products and Food, Hebei North University, Zhangjiakou 075000, China; 3 Institute of Quality Standard and Testing Technology, Beijing Academy of Agriculture and Forestry Sciences, Beijing 100097, China

**Keywords:** quinoa protein, antioxidant peptides, protease screening, peptide sequence identification, optimization of enzymatic hydrolysis

## Abstract

This study used locally sourced black, red, and white quinoa from Zhangjiakou as experimental materials to optimize the preparation process of quinoa antioxidant peptides and identify their sequence characteristics. Protein was extracted using the alkali-soluble acid precipitation method. The optimal hydrolytic enzyme was selected by comparing the hydrolysis effects of eight proteases. Enzymatic hydrolysis conditions were optimized using single-factor experiments and response surface methodology. After purification by Sephadex G-15 gel filtration chromatography, peptide sequences were identified by liquid chromatography–tandem mass spectrometry (LC-MS/MS) combined with De novo sequencing technology. The results showed that black quinoa and alcalase were identified as the optimal raw material and hydrolytic enzyme, respectively. The optimal enzymatic hydrolysis conditions were an enzyme dosage of 4000 U·g^−1^, hydrolysis time of 3.5 h, temperature of 42 °C, and pH 7.6, under which the average DPPH radical scavenging rate reached 86.61%. The purified G-1 fraction exhibited the highest antioxidant activity. A total of 1549 peptide sequences with confidence ≥90% were identified, among which peptides containing two or more hydrophobic amino acids accounted for 60.62%. This compositional feature is consistent with structural characteristics commonly associated with antioxidant peptides. This study provides a reference for the processing of quinoa-based functional ingredients.

## 1. Introduction

Quinoa (*Chenopodium quinoa* Willd.) is highly nutritious, with a protein content comparable to that of beef and a balanced amino acid composition. It is also rich in starch, lipids, minerals, vitamins, and bioactive compounds such as polyphenols, flavonoids, and saponins. It exhibits good in vitro antioxidant activity and meets human essential nutrient requirements, leading the Food and Agriculture Organization of the United Nations (FAO) to officially recommend it as a complete “whole nutrition food” most suitable for humans [1,2,3].

Excess reactive oxygen species (ROS) generated through metabolic processes can induce oxidative stress (OS), which is not only a key driver of aging and various chronic diseases but also causes oxidative deterioration, flavor degradation, and nutrient loss in foods, compromising food safety [4,5]. As natural antioxidants, antioxidant peptides can mitigate these issues and help maintain physiological balance, and may contribute to the mitigation of oxidative stress-related conditions, although this requires further investigation [6].

Previous studies have confirmed the potential of various proteases, such as papain, trypsin, alcalase, and bromelain, to hydrolyze quinoa protein into peptide fragments with demonstrated antioxidant activity [7,8,9,10]. Alcalase, in particular, has received attention for its high efficiency and its hydrolysates’ ability to activate intracellular antioxidant enzyme systems [9,10]. However, these investigations are often limited to one or two enzyme types and specific quinoa varieties, leaving a critical gap in our understanding of how protease specificity interacts with different quinoa cultivars to influence peptide bioactivity. Furthermore, the individual and interactive effects of key enzymatic parameters—enzyme dosage, time, temperature, and pH—on the antioxidant capacity of the resulting hydrolysates have not been systematically optimized. This lack of comprehensive data results in suboptimal preparation processes that may fail to harness the full functional potential of quinoa proteins. Most critically, the specific amino acid sequences responsible for the observed antioxidant activity remain largely uncharacterized. The ambiguity of the sequence–activity relationship significantly hinders the elucidation of their mechanism of action and constrains their application in functional food development.

The Zhangjiakou region, as one of China’s primary quinoa production areas, offers a valuable and regionally distinct resource for studying cultivar-specific protein functionality. Notably, different-colored quinoa varieties (black, red, and white) exhibit significant variations in protein composition and amino acid profiles, which may substantially influence their susceptibility to enzymatic hydrolysis and the bioactivity of the resulting peptides. This natural diversity provides a unique scientific opportunity to systematically compare the hydrolysis behavior and antioxidant potential across cultivars. Developing high-value products like quinoa antioxidant peptides from these locally sourced varieties thus presents not only an economic opportunity but also a scientific rationale for developing cultivar-specific processing protocols. However, while prior studies have confirmed the feasibility of using various proteases to generate antioxidant peptides from quinoa protein and have documented general antioxidant activity, three critical gaps remain unaddressed: (1) a systematic, head-to-head comparison of protease performance across different quinoa cultivars from the same production region is lacking; (2) the interactive effects of enzymatic parameters have not been comprehensively optimized for locally sourced varieties, which may lead to suboptimal bioactivity; and (3) the precise amino acid sequences responsible for antioxidant activity have not been identified, leaving the mechanism of action largely speculative. These unresolved issues collectively constrain the rational development of quinoa antioxidant peptides for functional food applications.

In light of the aforementioned scientific gaps, the selection of the most suitable protease for hydrolyzing proteins from these locally sourced varieties, the systematic optimization of hydrolysis conditions, and the comprehensive characterization of the active peptide sequences emerge as the pivotal scientific issues constraining the industrial application and mechanistic understanding of quinoa antioxidant peptides. The specificity of proteases leads to variable bioactivity; enzymatic parameters directly influence the yield and activity of the final product; and robust sequencing techniques are indispensable for elucidating their structure–activity relationships. Therefore, to systematically address these unresolved issues, this study utilizes three locally grown colored quinoa varieties (black, red, and white) from Zhangjiakou. We conducted a comprehensive investigation encompassing protein extraction, a comparative screening of eight proteases, and a response surface methodology-based optimization of enzymatic hydrolysis parameters. The resulting antioxidant peptides were subsequently purified and their sequences identified via LC-MS/MS with de novo sequencing. The overarching aims are to (1) establish an efficient and evidence-based preparation process for quinoa antioxidant peptides, (2) clarify the sequence features associated with their potent antioxidant activity, and (3) thereby provide a robust scientific basis for the deep processing of quinoa and the development of novel functional foods.

## 2. Materials and Methods

### 2.1. Materials

Quinoa was provided by Beimai Ecological Agriculture Co., Ltd., Guyuan County, Zhangjiakou, China. Bromelain, papain, acid protease, neutral protease, alcalase, trypsin, complex protease, flavourzyme, DL-serine, L-cysteine, 1,1-diphenyl-2-picrylhydrazyl (DPPH), 2,2′-azino-bis(3-ethylbenzothiazoline-6-sulfonic acid) (ABTS), o-phthalaldehyde, and Sephadex G-15 were obtained from Shanghai Ruiyong Biotechnology Co., Ltd., Shanghai, China. Disodium hydrogen phosphate was purchased from Xilong Scientific Co., Ltd., Shantou, China. Salicylic acid and anhydrous ethanol were supplied by Tianjin Huihang Chemical Technology Co., Ltd., Tianjin, China. n-Hexane was purchased from Shanghai Macklin Biochemical Technology Co., Ltd., Shanghai, China.

### 2.2. Main Instruments and Equipment

The main instruments and equipment used in this study were as follows: heating magnetic stirrer (C-MAG HS4), IKA, Staufen, Germany; UV-Vis spectrophotometer (TU-1900), Beijing Purkinje General Instrument Co., Ltd., Beijing, China; TGL-16 tabletop high-speed centrifuge, Sigma Centrifuge Co., Osterode am Harz, Germany; constant temperature water bath (DFD-700), Shanghai Shuli Instrument Co., Ltd., Shanghai, China; pH meter (PHSJ-4F), Shanghai INESA Scientific Instrument Co., Ltd., Shanghai, China; vortex mixer (MX-S), Scilogex, Rocky Hill, CT, USA; multimode microplate reader (iD3), Molecular Devices, San Jose, CA, USA; high-speed refrigerated centrifuge (HMX), Thermo Fisher Scientific, Waltham, MA, USA; vacuum freeze dryer (SCIENTZ-18N/A), Ningbo Xinzhi Biotechnology Co., Ltd., Ningbo, China; high-performance liquid chromatography system (Easy-nLC 1200) and electrospray ionization hybrid ion trap Orbitrap mass spectrometer, Thermo Fisher Scientific, Waltham, MA, USA.

### 2.3. Experimental Methods

#### 2.3.1. Preparation of Quinoa Antioxidant Peptides

##### Quinoa Protein Extraction

Quinoa grains with plump kernels and no mechanical damage were selected, rinsed three times with water, and dried in a drying oven at 45 °C. After crushing, the material was sieved through a 100-mesh screen and defatted using n-hexane at a solid-to-liquid ratio of 1:5. The defatted quinoa was then placed in a fume hood for 12 h to allow solvent evaporation, yielding defatted quinoa flour.

Quinoa protein was extracted using the alkali-soluble and acid-precipitation method, with reference to the method described by Ma Hongxin [11] and with minor modifications. The defatted quinoa flour was mixed with distilled water at a ratio of 1:12 (*w*/*v*), and the pH was adjusted to 11 using 1 mmol·L^−1^ NaOH. The mixture was extracted at 45 °C for 3 h, and then centrifuged at 5500 r/min for 20 min. The precipitate was discarded, and the supernatant was collected. The pH of the supernatant was adjusted to 4.5 using 1 mmol·L^−1^ HCl, and after standing for 30 min, the mixture was centrifuged at 5500 r/min for 20 min. The precipitate was collected, washed three times with deionized water, adjusted to pH 7.0, and dried for subsequent use.

##### Screening of Optimal Protease

The quinoa protein powder was added to distilled water to prepare a solution with a substrate concentration of 5% (*w*/*v*) for subsequent use. Bromelain, papain, flavourzyme, complex protease, trypsin, alcalase, neutral protease, and acid protease, each with an activity of 8000 U·g^−1^, were added at appropriate amounts. Enzymatic hydrolysis was carried out under their respective optimal pH and temperature conditions for 4 h (the optimal conditions for each protease are listed in Table 1). The enzymes were then inactivated by heating in a 95 °C water bath for 10 min, followed by rapid cooling to room temperature. The mixture was centrifuged at 5000 r/min for 15 min, and the supernatant was collected. The optimal protease was selected using DPPH radical scavenging rate as the primary indicator, with the degree of hydrolysis as a reference indicator.

##### Single-Factor Experiment

A single-factor experiment was conducted with the following baseline hydrolysis conditions: enzyme concentration of 6000 U·g^−1^, pH 8.5, temperature 40 °C, and time 1.5 h. Gradient experiments were designed for each parameter. The gradients for enzyme concentration were set at 3000, 4000, 5000, 6000, 7000, and 8000 U·g^−1^; for hydrolysis time: 1.5, 2.5, 3.5, 4.5, 5.5, and 6.5 h; for hydrolysis temperature: 35, 40, 45, 50, 55, and 60 °C; and for pH: 6.5, 7.5, 8.5, 9.5, 10.5, and 11.5. The effects of enzyme concentration, time, temperature, and pH on the hydrolysis of quinoa protein were investigated.

##### Response Surface Experiment

Based on the results of the single-factor experiment, a response surface methodology (RSM) experiment was designed using Design-Expert v8.0.6.1 (Stat-Ease Inc., Minneapolis, MN, USA). An experimental model was established with antioxidant activity (%) as the response value, and four significant factors—A (enzyme concentration, U·g^−1^), B (time, h), C (temperature, °C), and D (pH)—each at three levels coded as −1, 0, and +1, to optimize the enzymatic hydrolysis conditions. The response surface experiment design is presented in Table 2.

##### Determination of Degree of Hydrolysis

The method was adapted from Lin Qiu et al. [12] with minor modifications. Aliquots of 0, 100, 200, 300, 400, and 500 µL of serine standard solution were transferred into test tubes, and the volume was adjusted to 500 µL with deionized water. Then, 3 mL of OPA reagent was added to each tube, mixed thoroughly, and allowed to react for 2 min. The absorbance was measured at 340 nm. A standard curve was plotted with serine concentration as the horizontal axis and the measured absorbance as the vertical axis.

The wavelength was first adjusted to 340 nm. A 100 µL aliquot of the hydrolysate was taken and diluted with deionized water to bring the measured value within the range of the standard curve. Then, 500 µL of the diluted hydrolysate was transferred into a test tube, mixed with 3 mL of OPA reagent, and allowed to react for 2 min. The absorbance was measured. The average absorbance value was used to determine the C_Serine-NH2_ (mmol·L^−1^) from the standard curve. The degree of hydrolysis (DH) was calculated using Formula (1):

(1) $$W_{\mathrm{Serine-NH2}}=C_{\mathrm{Serine-NH2}}\;\times \;\frac{V\times N}{X\times P\%}\;h=(W_{\mathrm{Serine-NH2}}-\beta )/\alpha \;\mathrm{DH}=h/W_{\mathrm{Serine-NH2}}$$

where: W_Serine-NH2_ (mmol·L^−1^) represents the content of Serine-NH_2_ per gram of protein; X (g) is the sample mass; P (%) is the protein mass fraction in the sample; V (L) is the volume of the hydrolysate; N is the dilution factor of the hydrolysate; h (mmol·L^−1^) is the number of peptide bonds cleaved per gram of quinoa protein during hydrolysis; α and β are correction factors, taken as 1.00 and 0.40, respectively.

##### Determination of Antioxidant Activity

The DPPH radical scavenging assay is widely used for screening antioxidant activity of protein hydrolysates due to its simplicity and reproducibility. However, it measures chemical radical-scavenging activity in a cell-free system, which differs from physiological antioxidant effects. To provide a more comprehensive assessment, three complementary assays (DPPH, hydroxyl radical, and ABTS) were employed in this study, and blank corrections were applied in all measurements to minimize interference.

The quinoa antioxidant peptides obtained from hydrolysis were evaluated for their antioxidant activity by measuring DPPH radical scavenging rate, hydroxyl radical scavenging rate, and ABTS radical scavenging rate, and the optimal enzyme was selected based on comprehensive assessment. The samples used for antioxidant activity assays were the liquid supernatants collected directly after centrifugation of the enzymatic hydrolysates, without lyophilization or concentration. In the protease screening and single-factor experiments, all samples were tested at equal volumes (2 mL) of supernatant to evaluate the volumetric antioxidant activity of the hydrolysates. Since the peptide content in the supernatants may vary under different hydrolysis conditions, this comparison reflects the activity per unit volume of hydrolysate rather than the specific activity per unit mass of peptide.

1. DPPH radical scavenging rate

The method was adapted from Lu [13] with slight modifications. A 2 mL aliquot of the sample was mixed with 2 mL of DPPH solution (prepared in anhydrous ethanol), shaken thoroughly to ensure uniform mixing, and allowed to stand at room temperature in the dark for 30 min. The absorbance was measured at 517 nm (A_2_). Meanwhile, the absorbance of the blank group (anhydrous ethanol mixed with DPPH solution) was measured as A_0_, and the absorbance of the control group (sample solution mixed with anhydrous ethanol) was measured as A_1_. The DPPH radical scavenging rate was calculated using the following formula:

DPPH radical scavenging rate (%) = (1 − (A_2_ − A_1_)/A_0_) × 100 (2)

2. Hydroxyl radical scavenging rate

The method was adapted from Zhang Wenmin et al. [14] with slight modifications. A 1 mL aliquot of the sample was added to a test tube, followed by 1 mL of 6 mmol·L^−1^ FeSO_4_ solution and 1 mL of H_2_O_2_ solution. The mixture was shaken thoroughly to ensure uniform mixing and allowed to stand for 10 min. Then, 1 mL of 6 mmol·L^−1^ salicylic acid solution was added, and the mixture was incubated in a water bath at 37 °C for 30 min. After incubation, the absorbance was measured at 510 nm (A_i_). A blank solution was used as a control to measure the absorbance (A_0_). The hydroxyl radical scavenging rate was calculated using the following formula:

Hydroxyl radical scavenging rate (%) = (A_0_ − A_i_)/A_0_ × 100 (3)

3. ABTS radical scavenging rate

The method was adapted from Xie Jiahan et al. [15]. The ABTS^+^ stock solution was prepared by thoroughly mixing 7.4 mmol·L^−1^ ABTS^+^ solution and 2.6 mmol·L^−1^ potassium persulfate solution at a ratio of 1:1, and the mixture was allowed to stand in the dark for 12 h. The stock solution was then diluted with phosphate buffer (pH 7.4) until the absorbance reached 0.7 ± 0.02 at 734 nm, recorded as A_0_. A 0.2 mL aliquot of the sample was thoroughly mixed with 0.8 mL of ABTS working solution and allowed to stand in the dark for 6 min, after which the absorbance was measured at 734 nm (A_1_). The ABTS radical scavenging rate was calculated using the following formula:

ABTS radical scavenging rate (%) = (1 − A_1_/A_0_) × 100 (4)

#### 2.3.2. Purification of Quinoa Antioxidant Peptides

##### Gel Preparation

Sephadex G-15 was weighed into a beaker, and an adequate amount of distilled water was added. The mixture was heated in a boiling water bath for 5 h to ensure complete swelling. After swelling, the gel was washed 3–4 times by decantation to remove suspended fine particles from the gel surface, thereby preventing bubble formation or stratification within the column during packing.

##### Column Packing

The method was adapted from Zhang Lu et al. [16]. The chromatography column was vertically fixed using an iron stand, and the outlet was closed. Distilled water was used as the eluent. A small amount of absorbent cotton was placed at the bottom of the column, and eluent was added. The outlet was then opened to check for the absence of air bubbles before being closed again. The gel suspension was steadily poured into the column along a glass rod. Once the gel had settled to a height of approximately 2 cm at the bottom of the column, the outlet was opened, and the gel suspension was continuously added slowly until the gel deposition surface was about 5 cm from the top of the column, after which the outlet was closed. After the column bed was packed, eluent was added, the outlet was opened to allow the eluent to flow out at a constant rate, and the column bed was washed with 3–5 column volumes of eluent and equilibrated for 10 min.

##### Sample Loading and Elution

After the column bed was equilibrated, the outlet was opened to allow excess eluent to flow out at a constant rate. When the remaining liquid on the gel bed surface just covered the gel bed, the outlet was closed. A 2 mL sample was slowly applied in a circular motion along the inner wall of the chromatography column onto the gel bed surface using a micropipette. The outlet was then opened, and after the sample had completely entered the gel bed, 1–2 column volumes of eluent were added dropwise. Elution was continued until the components of the sample were fully separated, as indicated by the appearance of distinct peaks in the elution profile monitored by UV absorbance at 280 nm. The outlet was then opened, and after the sample had completely entered the gel bed, 1–2 column volumes of eluent were added dropwise for elution until the two bands were completely separated.

##### Collection of Samples

Collection was commenced immediately after the sample was loaded onto the column bed. The antioxidant activity of fractions from the same elution peak was evaluated by calculating the DPPH radical scavenging rate.

##### Gel Recovery

After sample collection, the chromatography column was repeatedly washed with 3–4 column bed volumes of distilled water. The column was then inverted, and the gel was blown out from the outlet using an earwash ball. The recovered gel was collected. If the gel was not to be used for an extended period, it was stored in a 60–70% ethanol solution at 4 °C in a refrigerator.

#### 2.3.3. Sequence Determination of Antioxidant Peptides

For sample preparation, an appropriate amount of the lyophilized sample was dissolved in 50 mM NH_4_HCO_3_, reduced with 10 mM dithiothreitol (DTT) at 56 °C for 1 h, and alkylated with 50 mM iodoacetamide (IAM) at room temperature in the dark for 40 min. The sample was then desalted using a self-packed desalting column and dried in a vacuum concentrator at 45 °C.

LC-MS/MS analysis was performed on an Easy-nLC 1200 system coupled with a Q Exactive™ Hybrid Quadrupole-Orbitrap™ mass spectrometer (Thermo Fisher Scientific, Waltham, MA, USA) equipped with a nano-electrospray ionization source. Peptide separation was carried out on a nano-LC column (150 μm i.d. × 150 mm) packed with Acclaim PepMap RPLC C18 (3 μm, 100 Å, Thermo Fisher Scientific, Waltham, MA, USA). The mobile phases consisted of 0.1% formic acid in water (phase A) and 0.1% formic acid in 80% acetonitrile (phase B). A 4 μL aliquot of each sample was loaded, and peptides were eluted at a flow rate of 600 nL/min using the following linear gradient: 4% to 8% B for 2 min, 8% to 28% B for 43 min, 28% to 40% B for 10 min, 40% to 95% B for 1 min, and maintained at 95% B for 10 min.

Mass spectra were acquired in positive ion mode using a data-dependent acquisition method. Full-scan MS spectra were recorded in the Orbitrap over a mass range of *m*/*z* 100–1500 at a resolution of 70,000 (at 400 *m*/*z*), with an automatic gain control (AGC) target of 3 × 10^6^ and a maximum injection time of 100 ms. The top 20 most intense precursor ions were selected for fragmentation using higher-energy collisional dissociation (HCD) with a normalized collision energy of 28%. MS/MS spectra were acquired in the Orbitrap at a resolution of 17,500 (at 400 *m*/*z*), with an AGC target of 1 × 10^5^ and a maximum injection time of 50 ms. Dynamic exclusion was enabled to exclude previously selected precursor ions.

The raw mass spectrometry data were processed using PEAKS Studio software (version 10.6, Bioinformatics Solutions Inc., Waterloo, ON, Canada) for de novo peptide sequencing. The following parameters were applied: fixed modification: carbamidomethylation (C); variable modifications: oxidation (M) and acetylation (N-term); enzyme specificity: none; maximum missed cleavages: 3; precursor mass tolerance: 20 ppm; and fragment mass tolerance: 0.02 Da. Peptide sequences with a confidence score ≥ 90% were considered for further structural analysis.

In PEAKS Studio software (version 10.6), peptide confidence scores are expressed as percentages and are derived from the De novo Score (also referred to as the Average Local Confidence, ALC). The ALC is calculated as the sum of the positional confidence scores for each amino acid residue in the peptide sequence divided by the total number of amino acids. The positional confidence score reflects the algorithm‘s confidence that a particular amino acid assignment is correct, based on the match between observed fragment ions and the predicted fragmentation pattern of the candidate sequence. A higher ALC score indicates a greater likelihood that the peptide sequence is correctly identified. Peptide sequences with a confidence score of ≥80% were initially retained, and only those with a confidence score of ≥90% were considered for further structural analysis.

## 3. Results

### 3.1. Results of Preparation of Quinoa Antioxidant Peptides

#### 3.1.1. Standard Curve of Serine

**Figure 1:**
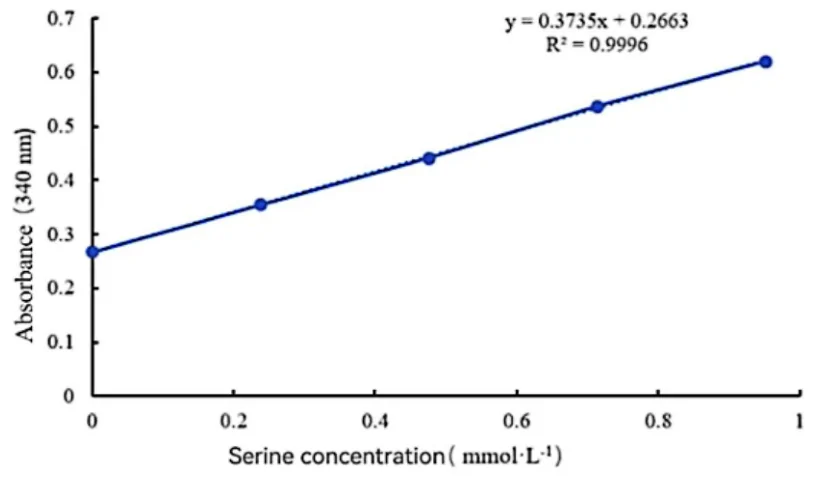
Standard curve of serine for the determination of degree of hydrolysis. Serine standards (0–500 µL) were reacted with o-phthalaldehyde (OPA) reagent for 2 min, and absorbance was measured at 340 nm. The linear regression equation was y = 0.3735x + 0.2663 (R^2^ = 0.9996). Data are presented as mean of triplicate measurements (*n* = 3); error bars are smaller than the symbols.

As shown in Figure 1, the serine concentration was positively correlated with the absorbance value, yielding the linear equation y=0.3735x+0.2663 with $R^{2}=0.9996$.

#### 3.1.2. Determination Results of Degree of Hydrolysis

**Figure 2:**
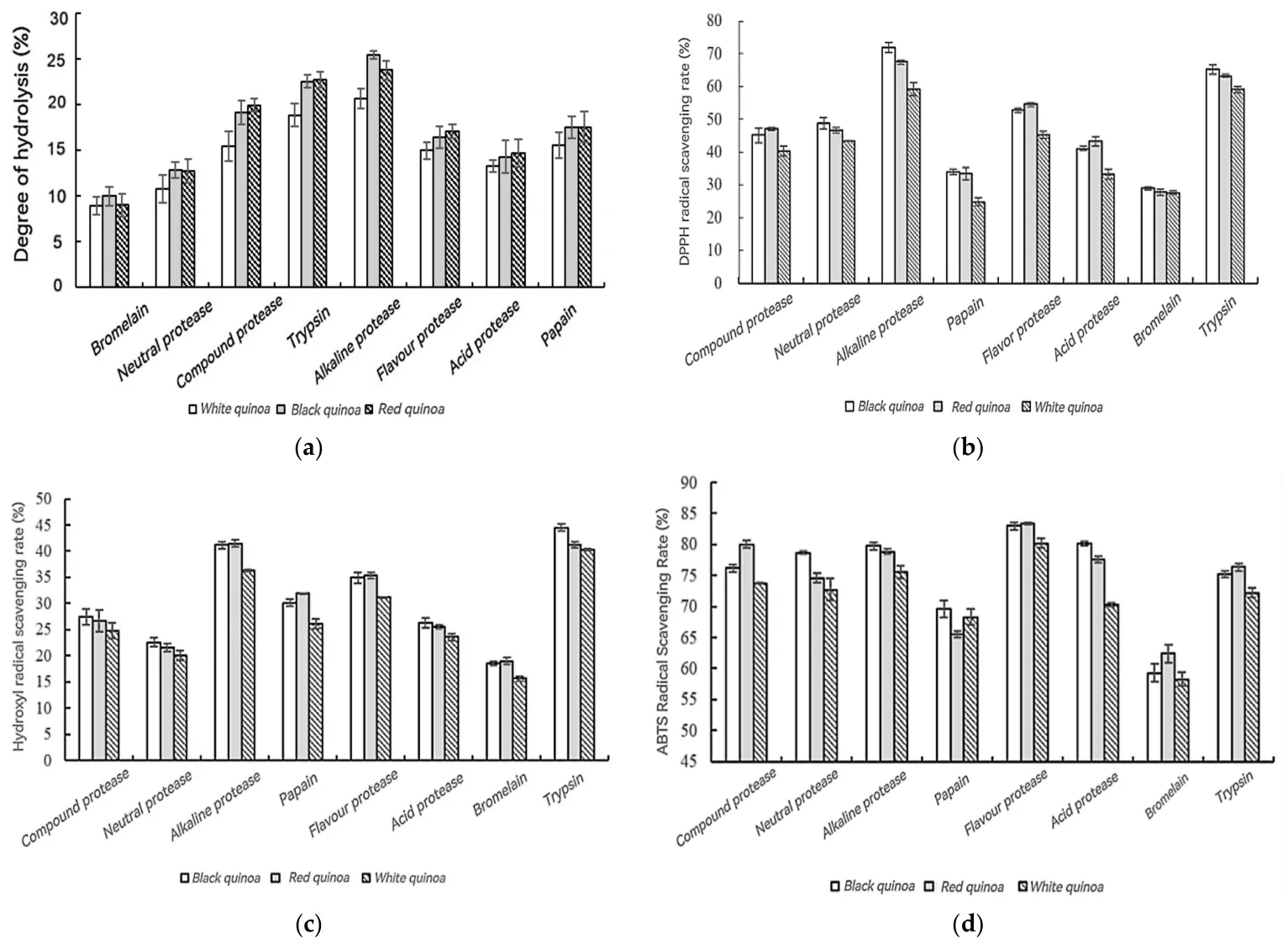
Effects of eight proteases on (**a**) degree of hydrolysis (DH), (**b**) DPPH radical scavenging activity, (**c**) hydroxyl radical scavenging activity, and (**d**) ABTS radical scavenging activity of protein hydrolysates from white, red, and black quinoa. Enzymatic hydrolysis was performed at each protease’s optimal pH and temperature for 4 h with an enzyme activity of 8000 U·g^−1^. DH was determined using the OPA method at 340 nm. DPPH scavenging was measured at 517 nm after 30 min in the dark; hydroxyl radical scavenging was measured at 510 nm after 30 min at 37 °C; ABTS^+^ scavenging was measured at 734 nm after 6 min in the dark. Data are presented as mean ± SD (*n* = 3).

As shown in Figure 2a, the degree of hydrolysis of quinoa protein varied among different proteases. Specifically, the black and red quinoa proteins exhibited higher degrees of hydrolysis under the action of alcalase, reaching (25.45 ± 0.71)% and (23.80 ± 0.67)%, respectively, whereas the lowest degrees of hydrolysis for the white, red, and black quinoa proteins were observed with bromelain, being (8.87 ± 0.62)%, (8.97 ± 0.75)%, and (9.98 ± 0.78)%, respectively. This phenomenon may be attributed to the close relationship between the cleavage sites of different proteases and the inherent specificity of hydrolysis, which also results in differences in the efficacy and activity of the resulting peptides [17]. Proteases from different sources have distinct characteristics, leading to significant differences in the degree of hydrolysis of the same protein. Throughout the enzymatic hydrolysis process, alcalase yielded the highest degree of hydrolysis.

#### 3.1.3. Screening Results of Optimal Protease

As shown in Figure 2b, among the eight proteases tested on the three quinoa protein varieties, the hydrolysate from black quinoa digested with alcalase exhibited the highest antioxidant activity, with a DPPH radical scavenging rate of (71.88 ± 1.54)%. In contrast, the white quinoa hydrolysate prepared with papain showed the lowest activity, with a DPPH radical scavenging rate of only (24.84 ± 1.24)%. This difference may be attributed to the fact that alcalase can effectively recognize specific amino acid sequences during quinoa protein cleavage, thereby generating a higher proportion of small peptides with potent antioxidant properties [18]. Furthermore, it is evident from the figure that antioxidant peptides derived from black and red quinoa consistently exhibited higher antioxidant activities than those from white quinoa.

As shown in Figure 2c, when the hydroxyl radical scavenging rate was used as the evaluation index, the black quinoa protein hydrolysate prepared with trypsin exhibited the highest antioxidant activity, with a value of (44.47 ± 0.66)%, followed by the red quinoa protein hydrolysate digested with alcalase, which showed a hydroxyl radical scavenging rate of (41.43 ± 0.60)%. No significant difference was observed between the trypsin-treated and alcalase-treated quinoa protein hydrolysates (*p* > 0.05). In contrast, the white quinoa protein hydrolysate hydrolyzed by bromelain showed the lowest activity, with a hydroxyl radical scavenging rate of only (15.63 ± 1.54)%. The hydroxyl radical is a highly reactive oxygen species with strong oxidizing properties, which can directly or indirectly induce lipid peroxidation and protein denaturation [19]. Furthermore, it is evident from the figure that antioxidant peptides derived from red and black quinoa consistently exhibited higher antioxidant activities than those derived from white quinoa.

As shown in Figure 2d, when the ABTS radical scavenging rate was used as the evaluation index, the black and red quinoa protein hydrolysates prepared with flavourzyme exhibited the highest antioxidant activities, with values of (82.99 ± 0.63)% and (83.37 ± 0.39)%, respectively. These were followed by the black quinoa protein hydrolysate digested with acid protease, the red quinoa protein hydrolysate prepared with complex protease, and the red and black quinoa protein hydrolysates digested with alcalase. However, no significant differences were observed among the hydrolysates obtained with different proteases in terms of ABTS radical scavenging activity (*p* > 0.05). The antioxidant activities of the resulting peptides varied to some extent depending on the enzyme used, which may be attributed to the intrinsic properties of the proteases, the specificity of different quinoa proteins, and the differences in peptide chain length and amino acid composition after hydrolysis.

Based on the comprehensive evaluation of the three radical scavenging indices and the degree of hydrolysis, black quinoa exhibited the best overall performance and was therefore selected as the optimal raw material for subsequent experiments. Regarding antioxidant activity, the alcalase hydrolysate showed the highest DPPH radical scavenging rate, while the trypsin hydrolysate performed best in hydroxyl radical scavenging, and the flavourzyme hydrolysate exhibited the highest ABTS radical scavenging rate. Nevertheless, no significant differences were found between trypsin or flavourzyme and alcalase in their hydroxyl and ABTS radical scavenging activities (*p* > 0.05). Moreover, alcalase achieved a higher degree of hydrolysis, is widely available, and is more cost-effective than trypsin. Therefore, alcalase was ultimately chosen as the protease for subsequent experiments.

#### 3.1.4. Results of Single-Factor Experiments

##### Effect of Enzyme Dosage on Antioxidant Activity and Degree of Hydrolysis

**Figure 3:**
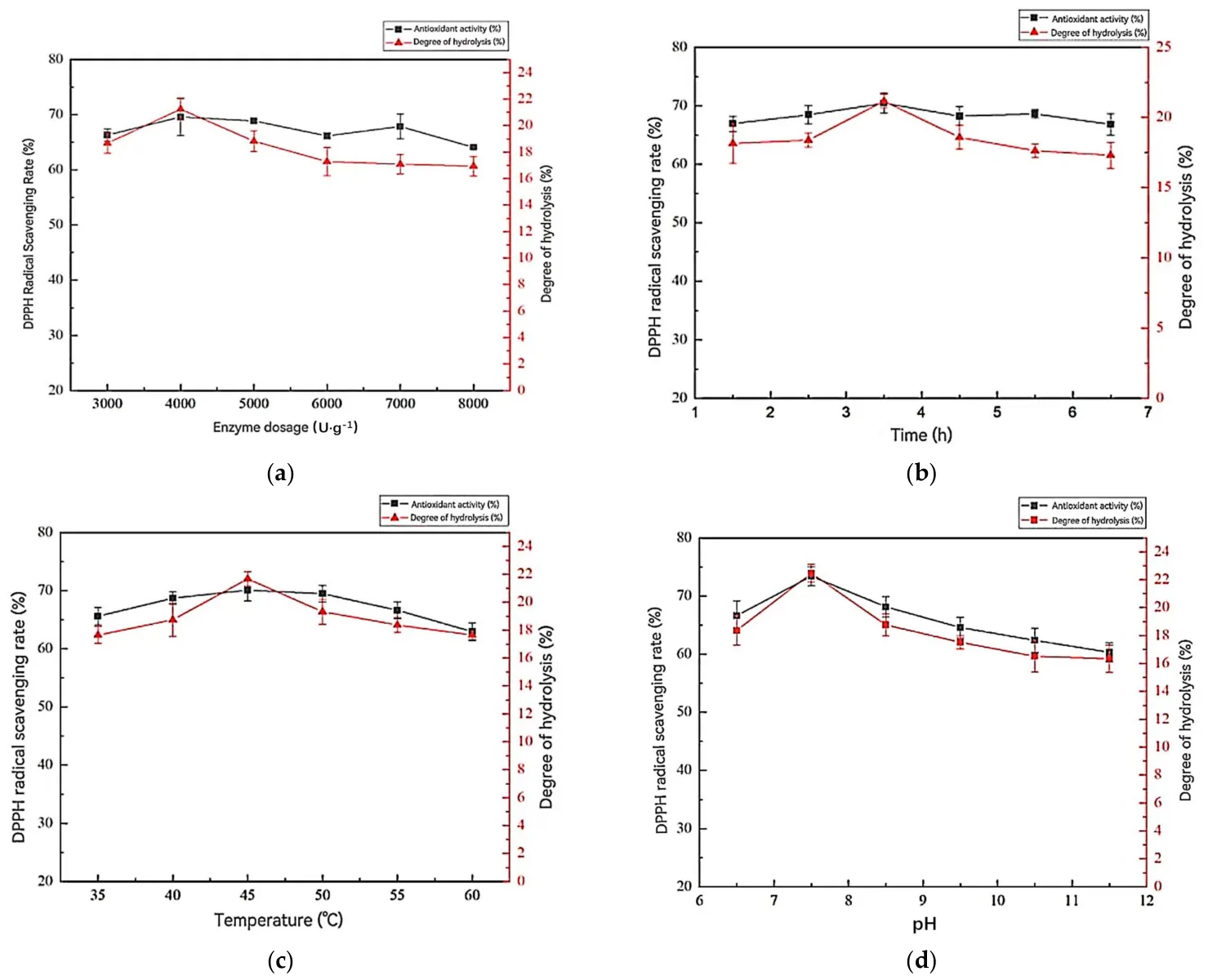
Effects of single factors on DPPH radical scavenging rate (bars) and degree of hydrolysis (lines) of black quinoa protein hydrolysates: (**a**) enzyme concentration (3000–8000 U·g^−1^), (**b**) hydrolysis time (1.5–6.5 h), (**c**) temperature (35–60 °C), and (**d**) pH (6.5–11.5). Unless otherwise specified, the baseline conditions were enzyme concentration 6000 U·g^−1^, pH 8.5, 40 °C, and 1.5 h. DPPH scavenging was measured at 517 nm after 30 min in the dark; DH was determined by the OPA method at 340 nm. Each value represents the mean ± SD (*n* = 3).

As shown in Figure 3a, both the antioxidant activity and the degree of hydrolysis of quinoa protein hydrolysates increased initially and then decreased with increasing enzyme concentration. The maximum values were observed at an enzyme concentration of 4000 U·g^−1^, with a DPPH radical scavenging rate of (69.57 ± 3.37)% and a degree of hydrolysis of (21.24 ± 0.83)%. Beyond this level, both the DPPH radical scavenging rate and the degree of hydrolysis gradually declined. During the increase in enzyme concentration, the enzyme and substrate became fully reacted, leading to the progressive accumulation of active peptides, which enhanced the antioxidant activity and degree of hydrolysis. However, after exceeding the optimal enzyme concentration, excessive enzyme levels caused over-degradation of intermediates, resulting in the decomposition of antioxidant peptides and consequently reducing both antioxidant activity and the degree of hydrolysis [20]. This trend is consistent with previous findings on the hydrolysis of quinoa protein by papain [21]. In summary, 4000 U·g^−1^ was selected as the optimal enzyme concentration for hydrolyzing quinoa protein.

##### Effect of Time on Antioxidant Activity and Degree of Hydrolysis

As shown in Figure 3b, both the antioxidant activity and the degree of hydrolysis of quinoa protein hydrolysates increased from 1.5 to 3.5 h. At 3.5 h, the DPPH radical scavenging rate and the degree of hydrolysis reached their maximum values, which were (70.43 ± 1.73)% and (21.15 ± 0.50)%, respectively. Beyond 3.5 h, both parameters began to decline. Notably, the decrease in the degree of hydrolysis is unusual, as the degree of hydrolysis would typically be expected to plateau with extended hydrolysis time. The initial increase up to 3.5 h is likely due to progressive enzymatic cleavage of quinoa proteins. For the subsequent decline, several explanations are plausible, including further cleavage of active peptides into smaller fragments with lower radical-scavenging activity, as suggested by previous reports [22], as well as partial protease autolysis or substrate exhaustion. However, because molecular weight distribution and compositional analyses were not performed at each time point in the present study, the precise cause of the observed decline remains to be confirmed. Future studies employing time-course monitoring of free amino nitrogen content using the o-phthalaldehyde method with enzyme-only controls, or sodium dodecyl sulfate-polyacrylamide gel electrophoresis analysis, would be valuable to verify whether peptide degradation or other factors account for this phenomenon. Based on these results, 3.5 h was selected as the optimal hydrolysis time for subsequent experiments.

##### Effect of Temperature on Antioxidant Activity and Degree of Hydrolysis

As shown in Figure 3c, both the DPPH radical scavenging rate and the degree of hydrolysis of quinoa protein hydrolysates increased initially and then decreased with rising temperature, which can be explained by the acceleration of the enzymatic reaction at elevated temperatures. At 45 °C, both indices reached their highest levels, with a DPPH radical scavenging rate of (70.07 ± 1.86)% and a degree of hydrolysis of (21.67 ± 0.49)%, after which they gradually decreased. Thus, the hydrolysis of quinoa protein is temperature-dependent. When the temperature exceeded the optimal reaction temperature, protease inactivation occurred, leading to incomplete enzymatic hydrolysis and reduced production of antioxidant peptides, which in turn lowered the DPPH radical scavenging rate and the degree of hydrolysis.

##### Effect of pH on Antioxidant Activity and Degree of Hydrolysis

As shown in Figure 3d, both the antioxidant activity and the degree of hydrolysis of quinoa protein hydrolysates reached their maximum values at pH 7.5, with a DPPH radical scavenging rate of (73.40 ± 1.61)% and a degree of hydrolysis of (22.47 ± 0.64)%. Beyond this pH, both parameters began to decline. This may be attributed to the intrinsic properties of the protease, its specific cleavage sites, and the dissociation states of enzyme and substrate molecules, as excessively high or low pH levels can lead to a reduction in enzyme activity [23].

#### 3.1.5. Optimization of Hydrolysis Conditions for Quinoa Antioxidant Peptides

##### Establishment and Significance Analysis of the Response Surface Regression Model

Based on the single-factor experiments, enzyme concentration, time, temperature, and pH were chosen as independent variables, with antioxidant activity, as the response. A Box–Behnken design was performed with Design-Expert 8.0.6.1 for the response surface experiments, and the results are shown in Table 3.

Using the response surface software to analyze the antioxidant activity results of quinoa protein hydrolysates, the quadratic regression model for enzyme concentration (A), time (B), temperature (C), and pH (D) was obtained as follows:

Y = 85.08 + 1.46A + 0.38B − 2.29C − 4.35D − 0.07AB + 2.46AC + 3.27AD + 0.36BC + 0.59BD − 8.46CD − 6.31A^2^ − 1.42B^2^ − 1.93C^2^ − 16.72D^2^ (5)

As shown in Table 4, the model was highly significant (*p* < 0.0001), while the lack-of-fit term was not significant (*p* = 0.1071 > 0.05), indicating a good fit of the equation. The coefficient of determination (R^2^) was 0.9726, and the adjusted R^2^ was 0.9453, suggesting that the model fitted the experimental data well and was reasonably established with statistical significance. Furthermore, the linear terms A, C, and D, as well as the quadratic terms A^2^, C^2^, and D^2^, all exhibited significant effects on the DPPH radical scavenging rate of quinoa protein hydrolysates.

##### Analysis of Interaction Effects in Response Surface Methodology

In the response surface 3D plots, the steepness of the curve reflects the degree of influence of the studied factors on the experimental results; the steeper the curve, the greater the influence. In the contour plots, a more elliptical shape of the contour lines indicates a significant interaction, whereas a less elliptical shape suggests a non-significant interaction [24]. As shown in the figures, the antioxidant activity of quinoa protein hydrolysates initially increased with increasing levels of each factor. After reaching the maximum value, the antioxidant activity declined with further increases in factor levels, confirming the existence of an optimal hydrolysis point in the experiment. Moreover, the optimal hydrolysis conditions were influenced not only by the individual factors—enzyme concentration, hydrolysis time, temperature, and pH—but also by their interactions.

**Figure 4:**
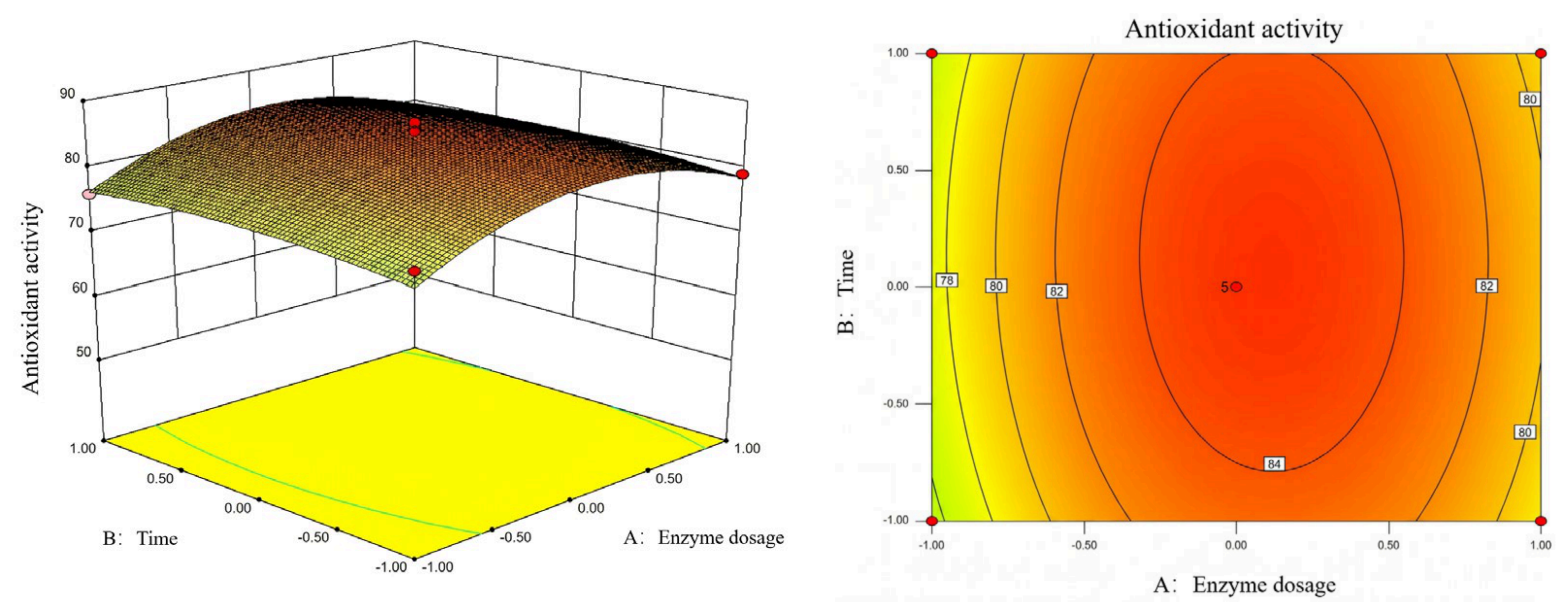
Y = f(A, B) Response surface and contour diagram. The color gradient indicates the magnitude of antioxidant activity; the numbers on contour lines represent the predicted response values; circles of different colors stand for experimental design points; green lines denote contour lines.

**Figure 5:**
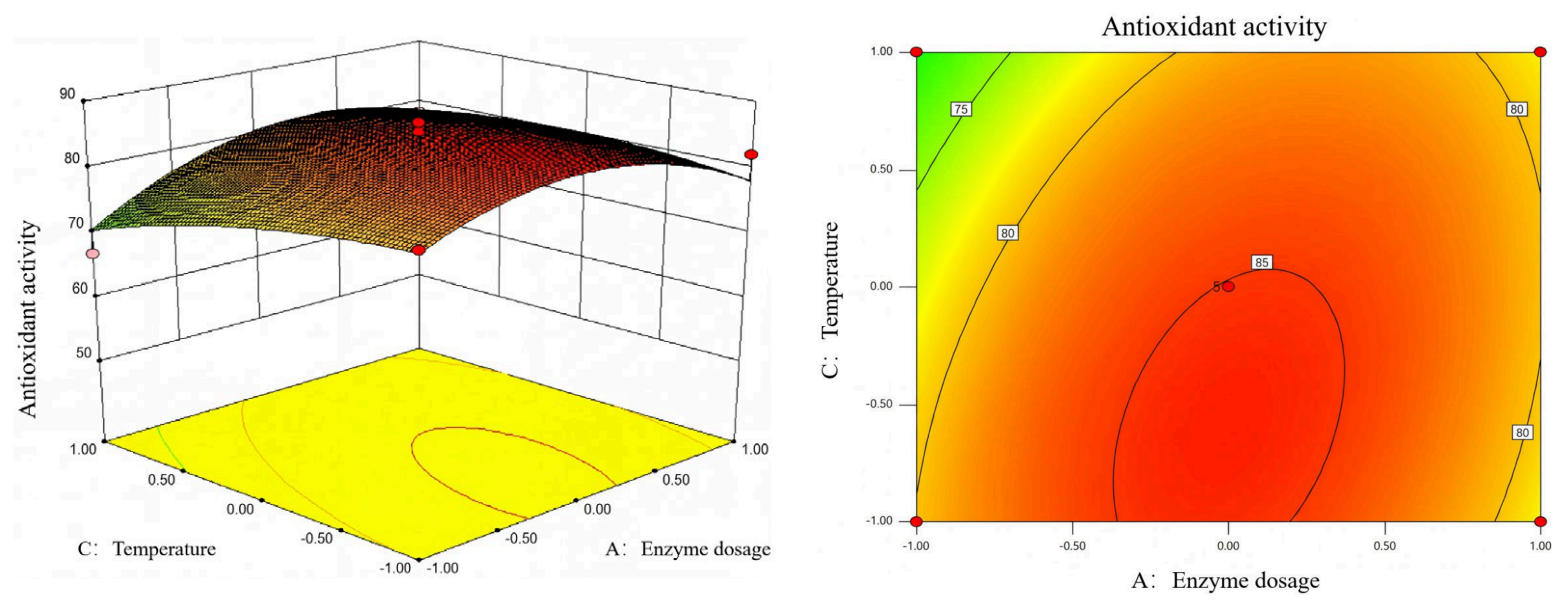
Y = f(A, C) Response surface and contour diagram. The color gradient indicates the magnitude of antioxidant activity; the numbers on contour lines represent the predicted response values; circles of different colors stand for experimental design points; green lines denote contour lines.

**Figure 6:**
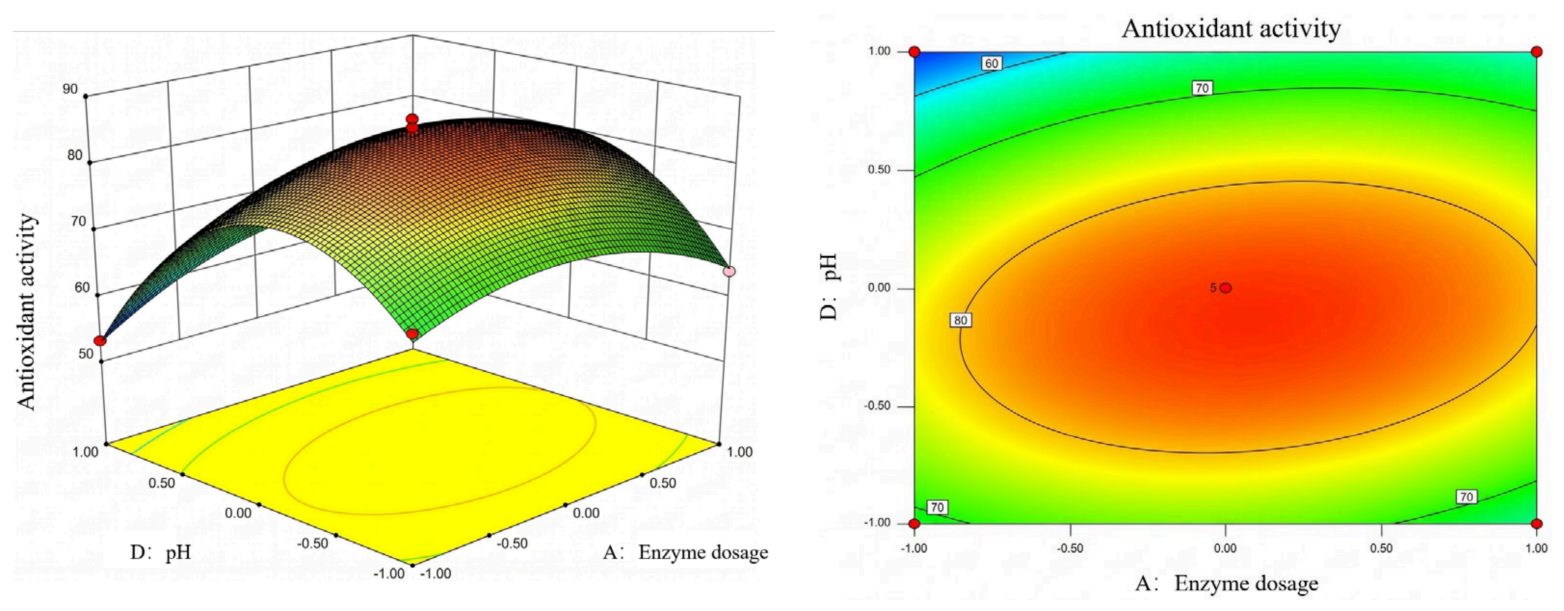
Y = f(A, D) Response surface and contour diagram. The color gradient indicates the magnitude of antioxidant activity; the numbers on contour lines represent the predicted response values; circles of different colors stand for experimental design points; green lines denote contour lines.

**Figure 7:**
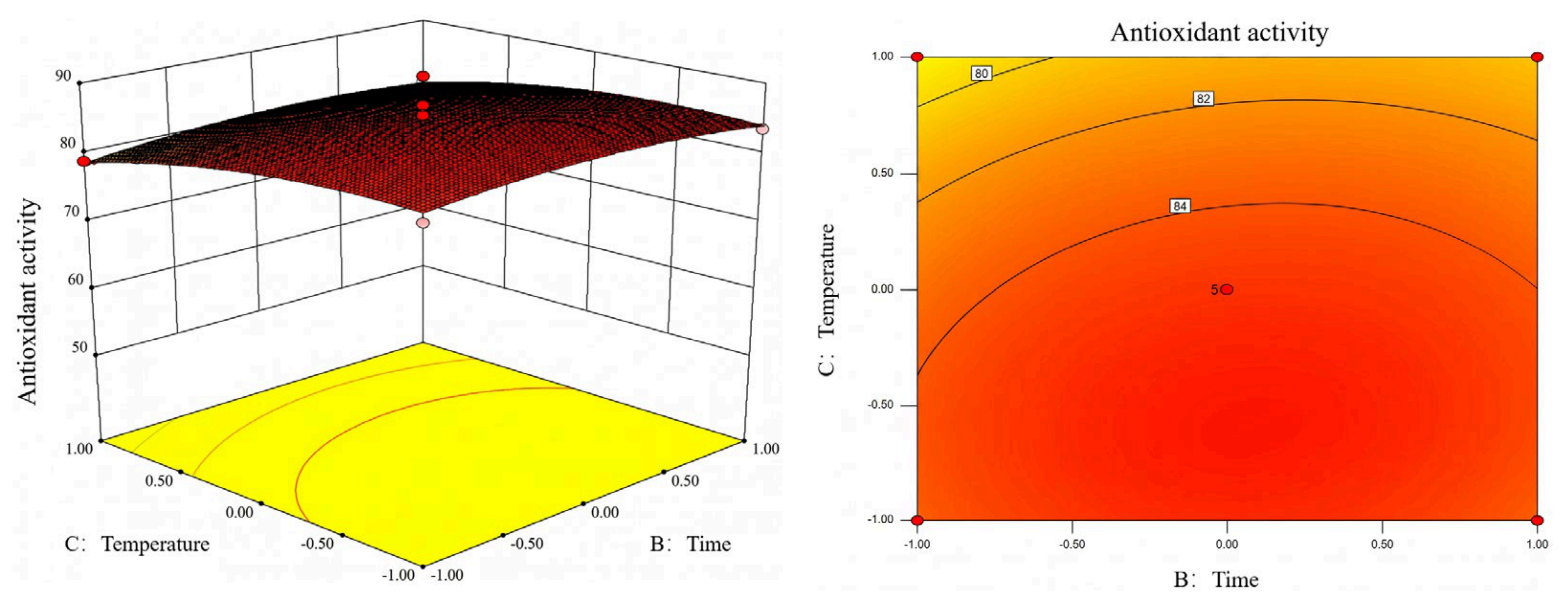
Y = f(B, C) Response surface and contours. The color gradient indicates the magnitude of antioxidant activity; the numbers on contour lines represent the predicted response values; circles of different colors stand for experimental design points; green lines denote contour lines.

**Figure 8:**
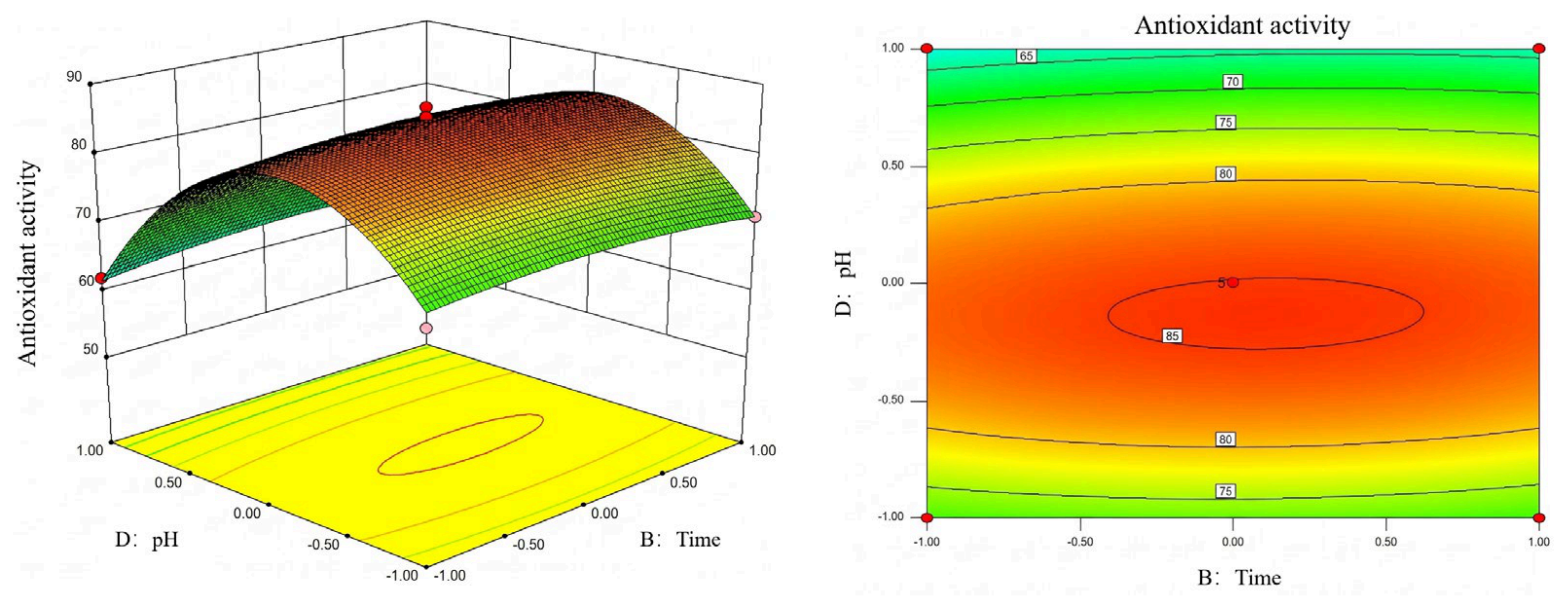
Y = f(B, D) response surface and contour diagram. The color gradient indicates the magnitude of antioxidant activity; the numbers on contour lines represent the predicted response values; circles of different colors stand for experimental design points; green lines denote contour lines.

**Figure 9:**
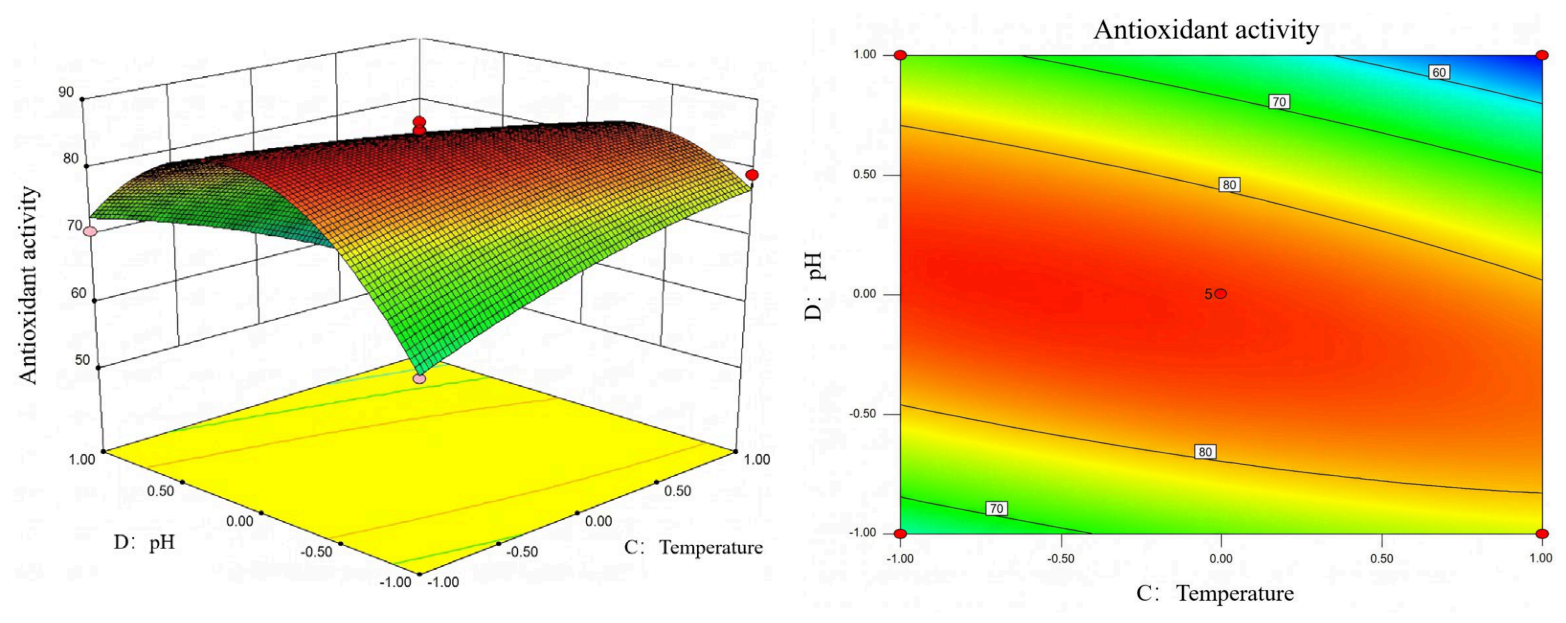
Y = f(C, D) response surface and contour diagram. The color gradient indicates the magnitude of antioxidant activity; the numbers on contour lines represent the predicted response values; circles of different colors stand for experimental design points; green lines denote contour lines.

As shown in Figure 4 Y = f(A, B), neither enzyme concentration nor hydrolysis time had a pronounced effect on the DPPH radical scavenging rate, and the nearly elliptical contour lines indicated a certain degree of interaction between them. As shown in Figure 5 Y = f(A, C), temperature exerted a greater influence on the DPPH radical scavenging rate than enzyme concentration, which is consistent with the ANOVA results. The contour plot was close to elliptical but not distinctly so, suggesting an interaction between the two factors. As shown in Figure 6 Y = f(A, D), based on the steepness of the plot, pH had a greater influence than enzyme concentration on the DPPH radical scavenging rate. The contour plot exhibited an elliptical shape, indicating a significant interaction between them. As shown in Figure 7 Y = f(B, C), the slight steepness of the plot indicated that neither factor had a pronounced effect on the DPPH radical scavenging rate; however, the plot showed a slightly greater tilt toward temperature, suggesting that temperature had a slightly greater impact on antioxidant activity than time. The contour lines were closer to circular than elliptical, indicating that the interaction between them was not significant. As shown in Figure 8 Y = f(B, D), pH had a greater influence on the DPPH radical scavenging rate than time, and the center of the contour plot was closer to an elliptical shape, implying an interaction between the two factors. As shown in Figure 9 Y = f(C, D), the steepness of the effect of pH on the DPPH radical scavenging rate was greater than that of temperature; therefore, the hydrolysis pH had a greater impact on antioxidant activity than temperature. The degree to which the contour lines approached an elliptical shape indicated a certain degree of interaction between them. In all response surface plots, a maximum point could be observed, indicating that interactions existed between each pair of influencing factors, albeit with varying levels of significance.

Based on the response surface results, the optimal hydrolysis conditions for quinoa antioxidant peptides were determined as follows: enzyme concentration of 3990 U·g^−1^, hydrolysis time of 3.55 h, temperature of 41.55 °C, and pH 7.55. Under these conditions, the predicted DPPH radical scavenging rate of the quinoa antioxidant peptides was 85.78%. To verify the accuracy of the model, the optimal conditions were rounded to practical values (enzyme concentration 4000 U·g^−1^, hydrolysis time 3.5 h, temperature 42 °C, and pH 7.6), and three replicate experiments were conducted. The average antioxidant activity of the quinoa antioxidant peptides obtained under these conditions was 86.61%, with an error of 0.83% compared to the predicted value. This indicated that the response surface model could reliably predict the optimal hydrolysis conditions for quinoa protein.

### 3.2. Purification of Antioxidant Peptides by Gel Filtration Chromatography

#### 3.2.1. Elution Profile

**Figure 10:**
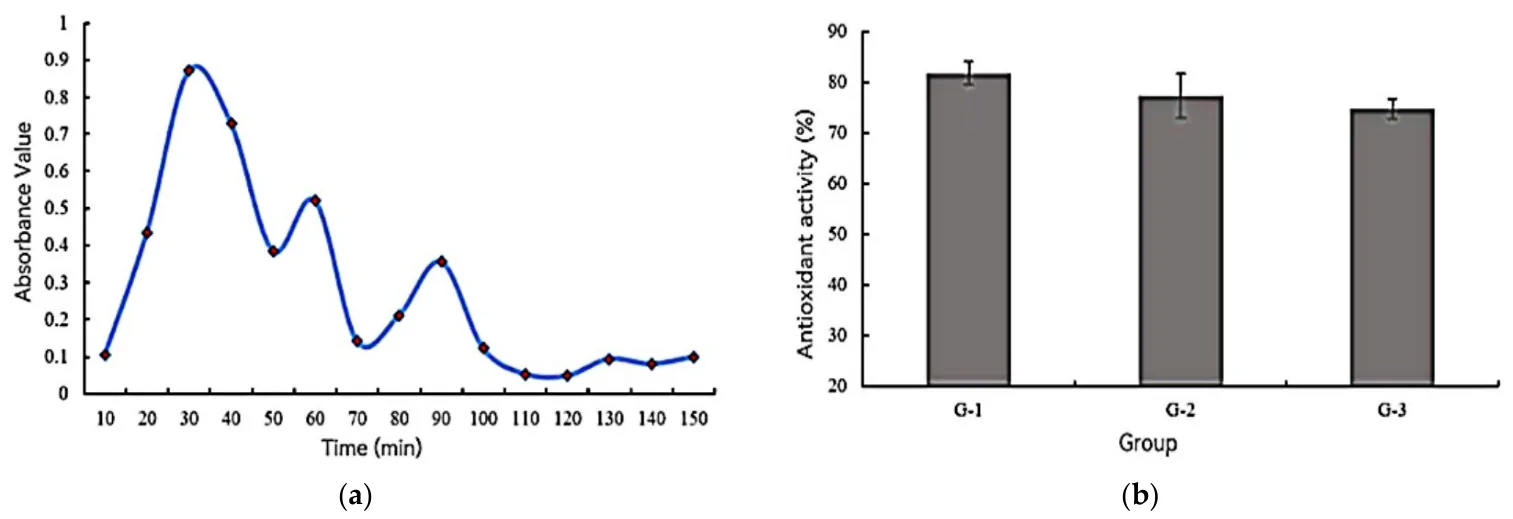
(**a**) Elution profile of quinoa antioxidant peptides purified by Sephadex G-15 gel filtration chromatography. The hydrolysate (2 mL) was loaded onto a column (1.6 × 60 cm) equilibrated with distilled water and eluted at a constant flow rate (0.5 mL/min). Absorbance was monitored at 280 nm; three major peaks (G-1, G-2, G-3) were collected according to elution time. (**b**) DPPH radical scavenging activities of the three pooled fractions (G-1 to G-3). Each fraction was tested at the same peptide concentration (1 mg/mL) in 2 mL of sample, and DPPH scavenging was measured at 517 nm after 30 min in the dark. Data are presented as mean ± SD (*n* = 3). Fraction G-1 exhibited the highest activity and was selected for further LC-MS/MS analysis.

As shown in Figure 10a, the quinoa antioxidant peptide hydrolysate was purified by gel filtration chromatography, yielding three elution peaks. The absorbance was measured at 280 nm, and the elution profile was plotted with collection time on the *x*-axis and absorbance on the *y*-axis. The peaks were designated as G-1, G-2, and G-3 according to their elution order.

#### 3.2.2. DPPH Radical Scavenging Activity of Different Fractions of Quinoa Antioxidant Peptides

After purification by gel filtration chromatography, three elution peaks were collected at approximately 30, 60, and 90 min, respectively. The antioxidant activities of the three collected fractions were evaluated using the DPPH radical scavenging rate as the index. As shown in Figure 10b, the antioxidant activities of the three purified fractions differed significantly. Among them, fraction G-1 exhibited the highest antioxidant capacity, with a DPPH radical scavenging rate of (81.74 ± 2.24)%, while fractions G-2 and G-3 showed DPPH radical scavenging rates of (77.26 ± 4.38)% and (74.61 ± 1.93)%, respectively, both lower than that of G-1. Therefore, the first elution peak (G-1) was collected and lyophilized for subsequent mass spectrometry identification. Notably, the antioxidant activity did not increase with increasing purity; rather, it showed a decreasing trend. This may be attributed to the fact that the unpurified quinoa antioxidant peptide hydrolysate contained a mixture of quinoa proteins and peptides, which together contributed to a higher overall antioxidant level, whereas the purified peptides did not exhibit a strictly positive correlation with purity [25].

### 3.3. Sequence Identification of Antioxidant Peptides

The lyophilized quinoa antioxidant peptides obtained after purification were subjected to reduction, alkylation, and desalting, and the processed samples were then analyzed by liquid chromatography-tandem mass spectrometry (LC-MS/MS). Subsequent de novo sequencing analysis yielded numerous peptide sequences, all with molecular weights ≤1500 Da, and the majority of which had molecular weights <1000 Da. This indicated that the peptides in the G-1 fraction purified by Sephadex G-15 gel filtration chromatography were of relatively small molecular size, which is consistent with the notion that peptides can effectively scavenge free radicals and exert their full antioxidant activity only when they possess small molecular weights [26].

Among the 20 amino acids, the hydrophobic amino acids—leucine (Leu/L), valine (Val/V), glycine (Gly/G), proline (Pro/P), methionine (Met/M), phenylalanine (Phe/F), tryptophan (Trp/W), alanine (Ala/A), and isoleucine (Ile/I)—can exert antioxidant activity by scavenging free radicals and increasing solubility in aqueous and lipid media. The classification of these amino acids as hydrophobic follows the widely used biochemical convention based on side-chain polarity and hydropathy scales. Glycine, despite having the smallest side chain (a single hydrogen atom), is conventionally grouped with non-polar hydrophobic amino acids due to its limited polarity and its tendency to participate in hydrophobic interactions during protein folding. However, it should be noted that glycine‘s classification can vary across different hydropathy scales, as its minimal side chain confers amphiphilic properties that allow it to exist in various environments. In addition, neutral, basic, and acidic amino acids can also contribute to antioxidant effects through their respective properties [27]. Tyrosine (Tyr/Y), Trp, Phe, and histidine (His/H) are aromatic amino acids that have also been demonstrated to possess good antioxidant activity [28]. In the de novo sequencing analysis, a total of 3393 peptide sequences with a confidence level above 80% were obtained, among which 1549 peptides had a confidence level higher than 90%, indicating a high accuracy of the identification results. The complete list of all identified peptide sequences is provided in the Supplementary Materials (Figure S1). Based on the amino acid sequences contained in the peptides, the 1549 peptides with confidence ≥ 90% were classified into five categories. As shown in Table 5, all peptide sequences contained hydrophobic amino acids. Only two peptides contained a single hydrophobic amino acid, accounting for approximately 0.13% of the total. A total of 87 peptides consisted entirely of hydrophobic amino acids, accounting for 5.62%. Peptides containing both hydrophobic and aromatic amino acids numbered 491, accounting for 31.70%. Peptides containing two or more hydrophobic amino acids numbered 939, accounting for 60.62%. There were 30 peptides composed solely of hydrophobic and aromatic amino acids, accounting for 1.93%. This compositional analysis demonstrates that the peptides identified in the G-1 fraction are predominantly characterized by hydrophobic amino acid residues, either alone or in combination with aromatic residues. Given that such structural features have been widely associated with antioxidant activity in previous peptide studies [27,28], the present results suggest that the G-1 fraction’s strong DPPH radical scavenging activity (81.74%) may be attributable, at least in part, to the abundance of peptides bearing these residue types. However, it is important to emphasize that the observed antioxidant activity was measured for the G-1 fraction as a whole—a complex mixture of 1549 peptide species—and does not confirm that each individual peptide within this mixture is itself an active antioxidant. The specific contribution of any single peptide to the overall activity remains to be determined through further peptide synthesis and activity verification.

Among the peptides with a confidence level above 90%, 20 sequences were selected for further structural analysis based on their hydrophobic amino acid content and similarity to previously reported antioxidant peptide fragments (Table 6). For example, sequences ASVGFKA, LLKAL, KMTTVH, TSPVHYE, and FDFHEK contain dipeptide motifs (KA, VH, HE) that have been reported in active antioxidant peptides [29]. Additionally, several sequences (VVGF, LLGPAL, LAVGL, MFGAP, YLPLL, VLPGVVGF, LVAF, FAYPGLL, LVGLF, FMAM) consist entirely of hydrophobic amino acids, while others (MPAGVAHW, LLPH, WVAL, PAGVAH, LVVLGH) contain both hydrophobic and aromatic residues. These structural features are consistent with the physicochemical properties commonly associated with antioxidant peptides, suggesting that these 20 peptides may serve as promising candidates for antioxidant activity. However, it must be emphasized that this prediction is based on structural inference only; the actual antioxidant activity of each individual peptide requires experimental confirmation through chemical synthesis followed by in vitro activity assays. Notably, some of these peptide sequences showed high similarity to previously reported quinoa peptides with ACE inhibitory activity, suggesting that they may possess multiple bioactivities simultaneously. Moreover, the content of hydrophobic amino acids in the peptides was positively correlated with their antioxidant activity [30,31].

## 4. Discussion

### 4.1. Screening of Proteases

Quinoa, as a cereal grain with high nutritional value, is rich in carbohydrates, lipids, and trace elements, and possesses a unique amino acid composition [32]. Its derived peptides have been reported to exhibit multiple bioactivities as evaluated by in vitro assays. However, it is important to distinguish between the chemical radical-scavenging activity measured in cell-free systems—such as the DPPH, hydroxyl radical, and ABTS assays employed in the present study—and the distinct concept of physiological antioxidant effects, which involve complex biological processes including enzyme regulation, signal transduction, and metabolic pathways. In the food industry, quinoa is predominantly used in its whole-grain form, whereas its application in the form of bioactive peptides has received less attention. Therefore, the development of quinoa antioxidant peptides for food applications holds considerable significance for research and industrial exploitation. In this study, eight proteases, three colored quinoa varieties, and three antioxidant activity assays were employed to screen for the optimal protease for the preparation of quinoa antioxidant peptides.

Eight different proteases, including complex protease, neutral protease, alcalase, papain, acid protease, flavourzyme, and bromelain, were used to hydrolyze quinoa protein for the preparation of quinoa antioxidant peptides. Using antioxidant activity and degree of hydrolysis as evaluation indices, the quinoa antioxidant peptides prepared from black quinoa hydrolyzed by alcalase exhibited superior antioxidant activity. All quinoa proteins digested by different proteases generated peptides with antioxidant activity; however, the specificity of each protease resulted in differences in the types and quantities of the resulting peptides. Therefore, hydrolysis of the same or different protein substrates with different proteases yielded varying antioxidant activities. Furthermore, alcalase preferentially cleaves peptide bonds at the carboxyl side of hydrophobic amino acids, which may lead to its hydrolysates containing abundant free hydrophobic amino acids and terminal hydrophobic residues. It is hypothesized that hydrophobic residues including Leu, Tyr and Met may improve radical-scavenging capacity via interactions with free radicals, and the hydrolysate produced under this hydrolysis pathway also presented relatively mild bitterness [33]. Previous studies have reported that alcalase is a suitable enzyme for the hydrolysis of plant proteins [34,35]. In the present study, among the eight proteases tested, alcalase exhibited the highest degree of hydrolysis and superior DPPH radical scavenging activity for black quinoa protein extracted by the alkali-soluble acid-precipitation method. This observation is consistent with previous reports on the efficacy of alcalase for plant protein hydrolysis [34,35]. Moreover, quinoa peptides prepared by alcalase hydrolysis retained significant antioxidant activity even after simulated gastrointestinal digestion in vitro [34,35]. In addition, the combined use of flavourzyme and alcalase can increase peptide diversity, and high-pressure pretreatment can further enhance the hydrolysis efficiency and product activity [36].

Alcalase is active when the substrate is neutral or alkaline, and current sequence identification has confirmed that alcalase is primarily a serine alkaline protease [37]. Both neutral and alkaline proteases possess a nucleophilic serine residue at their active sites. This type of protease requires aspartate and histidine residues, which are linked to the serine residue at the active site to form a catalytic triad, and the catalytic reaction generally proceeds under neutral or alkaline conditions, with an optimal pH range of 7.0–11.0 [38]. Wang Xin et al. [39] used alcalase to hydrolyze soybean protein and evaluated the antioxidant activity by measuring the ferric reducing power. Under the optimal hydrolysis conditions of temperature 72 °C, substrate concentration 9%, enzyme-to-substrate ratio (E/S) 1%, pH 7.0, and hydrolysis time 5 h, the highest antioxidant activity and degree of hydrolysis were achieved. Zhang Wenmin et al. [14] prepared small-molecular-weight peptides from flaxseed meal and identified the most antioxidant-active fractions using high-performance liquid chromatography–tandem mass spectrometry. Their results showed that the low-molecular-weight peptides prepared with alcalase exhibited superior antioxidant activity compared to those prepared with neutral protease and trypsin.

Among the three quinoa varieties, a comprehensive evaluation based on three antioxidant indices revealed that dark-colored quinoa exhibited higher antioxidant activity than light-colored quinoa. This finding is consistent with the report by Maldonado-Alvarado P et al. [40], who demonstrated that germination, following desaponification, soaking, and germination, could increase the content of proteins and other trace elements in quinoa, as well as enhance the nutritional components and phytase activity. Chen Yisheng et al. [41] also demonstrated that the in vitro antioxidant capacity of all three quinoa varieties increased after germination, with white quinoa showing a smaller magnitude of change compared to red and black quinoa. In the rinsing stage of this study, black quinoa germinated more rapidly than the other two varieties. During the early drying stage, the suitable temperature and the residual moisture after rinsing promoted the germination of quinoa, with black quinoa exhibiting a significantly higher germination rate than the other two colored varieties. In the antioxidant activity assays, black quinoa showed slightly higher antioxidant activity than red quinoa and significantly higher activity than white quinoa. Based on the comprehensive evaluation of the experimental results, black quinoa was selected as the raw material for subsequent experiments. Differences in protein constitution and amino acid profiles among three colored quinoa cultivars may serve as one core factor contributing to divergent antioxidant performance of their enzymatic hydrolysates [42].

Among the three antioxidant activity assays, the antioxidant activities of the quinoa antioxidant peptides prepared from the same colored quinoa with the same protease showed certain variations. Taking black quinoa hydrolyzed by alcalase as an example, the antioxidant activities determined by DPPH radical scavenging rate, hydroxyl radical scavenging rate, and ABTS radical scavenging rate were (71.88 ± 1.54)%, (41.15 ± 0.40)%, and (87.48 ± 0.38)%, respectively. For the antioxidant peptides prepared from the same colored quinoa protein hydrolyzed by the same protease, the differences in antioxidant mechanisms may lead to variations in the evaluation of their antioxidant activity. In addition, the scavenging rates of ABTS and DPPH radicals were higher than that of hydroxyl radicals in this study, which may be attributed to the further hydrolysis of some peptides capable of scavenging these two radicals by exopeptidases [43]. Since the DPPH radical scavenging rate is one of the most commonly used indicators for antioxidant activity determination, with wide applicability and good stability, it was adopted as the reference index for the subsequent single-factor experiments.

In the determination of the degree of hydrolysis, the antioxidant activity did not show a completely positive correlation with the degree of hydrolysis. This is consistent with the findings of Latorres et al. [44], who reported that although the degree of hydrolysis is to some extent related to the antioxidant activity, this correlation is not absolute or completely consistent. This lack of perfect correlation is potentially attributable to discrepancies in protease cleavage specificity, intrinsic structural features of quinoa protein substrates, and the divergent amino acid sequences of generated peptide fragments.

To contextualize the novelty of the present work, it is instructive to compare the antioxidant activity of the obtained quinoa peptides with previously reported counterparts. Gu et al. [35] reported that alcalase-hydrolyzed quinoa peptides exhibited strong DPPH radical scavenging activity, while Guo Huimin et al. [45] found that oat peptides prepared with alcalase showed an IC_50_ of 9.112 mg·mL^−1^ for DPPH radical scavenging. In contrast, the purified G-1 fraction in this study achieved a DPPH scavenging rate of 81.74% under the optimized conditions, indicating a highly competitive antioxidant capacity. More importantly, beyond activity evaluation, the present work provides a comprehensive identification of 1549 peptide sequences with confidence ≥ 90% and reveals that 60.62% of these peptides contain two or more hydrophobic amino acids—a structural feature closely associated with antioxidant potential. This sequence-level characterization, combined with the systematic comparison of multiple proteases and quinoa cultivars, offers new insights into the preparation and mechanism of quinoa antioxidant peptides, thereby underscoring the novelty and significance of this study.

### 4.2. Optimization of Preparation Conditions for Quinoa Antioxidant Peptides

In this study, alcalase was selected as the protease for hydrolyzing quinoa protein to prepare antioxidant peptides, and the influencing factors—temperature, pH, enzyme concentration, and time—were investigated with particular attention to their individual effects. Since alcalase was chosen as the protease, the pH was maintained within the range of 7–11 to allow effective catalysis. In addition, excessively high temperatures could partially inactivate the enzyme. Only when each factor meets the requirements for quinoa protein hydrolysis can the resulting quinoa antioxidant peptides exhibit satisfactory antioxidant activity.

Based on the single-factor experiments, response surface methodology was employed to optimize the preparation conditions of quinoa antioxidant peptides. Ultrasound pretreatment can induce conformational unfolding of quinoa protein, thereby enhancing the hydrolysis efficiency and the antioxidant activity of the resulting products [46]. Under the optimal conditions, the antioxidant peptides obtained from three replicate experiments exhibited an average DPPH radical scavenging rate of 86.61%, with an error of 0.83% compared to the model-predicted value, indicating that the model could adequately reflect the preparation conditions of quinoa antioxidant peptides.

Enzymatic hydrolysis of proteins is currently one of the most widely used approaches for obtaining bioactive peptides, and response surface software can be employed to design experimental protocols and optimize process conditions through experimental results and model predictions. Chen Min et al. [46] used alcalase to prepare oat peptides and evaluated their antioxidant activity using DPPH and hydroxyl radical scavenging rates as indices. Through response surface methodology, the optimal hydrolysis conditions were obtained, under which the oat peptides exhibited the highest antioxidant activity, with an IC_50_ value of 9.112 mg·mL^−1^ for DPPH radical scavenging and 3.062 mg·mL^−1^ for hydroxyl radical scavenging. Xia Zhen et al. [47] used pepsin to hydrolyze selenium-enriched oyster protein for the preparation of selenium-enriched oyster peptides. The optimal enzymatic hydrolysis conditions, optimized by single-factor and response surface experiments, were determined as follows: time 4 h, temperature 37 °C, enzyme-to-substrate ratio 0.5%, pH 1.0, and substrate concentration 0.07 g·mL^−1^. Under these conditions, the prepared selenium-enriched oyster peptides exhibited good antioxidant activity and showed a significant protective effect against oxidative damage in HepG2 cells in a dose-dependent manner.

The preparation of active peptides by protease hydrolysis and subsequent activity evaluation is currently a research hotspot. Different proteases hydrolyze target substrates with varying cleavage sites, action characteristics, and optimal reaction conditions, leading to different choices of the most suitable protease across studies. Therefore, the selection of the most appropriate enzyme should be based on a comprehensive consideration of the experimental conditions and various influencing factors.

### 4.3. Purification Results of Quinoa Antioxidant Peptides

Size-exclusion chromatography, including gel filtration chromatography, separates molecules based on their molecular weight by selecting appropriate gel media. During elution, large molecules are excluded from the gel pores and elute earlier, while small molecules enter the pores and elute later [48,49]. This method is simple to operate, highly reproducible, and causes minimal denaturation of collected samples, making it a suitable choice for peptide purification. Sephadex G-15 was selected for this study due to its compatibility with the molecular weight range of the quinoa peptides generated (expected <1500 Da based on LC-MS/MS results).

In this study, Sephadex G-15 gel filtration chromatography was employed to separate and purify quinoa antioxidant peptides, yielding three purified fractions with distinct elution peaks. Their antioxidant activities were evaluated using the DPPH radical scavenging rate as the index. The G-1 fraction collected at approximately 30 min exhibited the strongest antioxidant activity, reaching (81.74 ± 2.24)%. The antioxidant activity of the purified peptides was slightly lower than that before purification, which is consistent with the findings of Ma Meng et al. [25], who reported that the antioxidant activity of tartary buckwheat peptides decreased after purification despite increased purity. It is proposed that crude hydrolysates contain a complex mixture of peptides, oligopeptides, free amino acids, endogenous phenolics and residual intact proteins that may exert synergistic antioxidant effects. During gel separation, partial co-existing active components may be separated from the target peptide fraction, which could partly explain the slight decline in DPPH activity of purified G-1 fraction [50].

Notably, the G-1 fraction, which eluted earliest from the Sephadex G-15 column and therefore is expected to contain peptides with the largest molecular sizes among the three fractions, exhibited the highest DPPH radical scavenging activity (81.74 ± 2.24)%. This observation warrants further interpretation, as it appears to contrast with the commonly held view that smaller peptides generally possess stronger antioxidant activity due to more efficient access to radical sites. However, the relationship between peptide molecular weight and antioxidant activity is not always straightforward and depends on multiple factors, including amino acid composition, sequence arrangement, and the specific radical-scavenging assay employed. In the present study, the compositional analysis of the G-1 fraction revealed a high abundance of hydrophobic and aromatic amino acid residues (Table 5), which are known to enhance radical-scavenging capacity by facilitating interactions with lipid radicals and serving as hydrogen donors. It is plausible that the relatively larger peptides within the G-1 fraction, despite their size, contain a higher density or more favorable arrangement of these active residues, resulting in a cumulative antioxidant effect that exceeds that of the smaller peptides in the G-2 and G-3 fractions. Additionally, synergistic interactions among multiple peptide species within the G-1 fraction may contribute to its overall activity. Nevertheless, further fractionation and activity-guided characterization of the G-1 subcomponents would be necessary to identify the specific peptides responsible for its potent radical-scavenging activity and to elucidate the precise structure–activity relationships involved.

### 4.4. Peptide Sequence Identification

De novo sequencing does not rely on any reference sequence for target detection. Compared with other peptide sequencing methods, this approach enables comprehensive determination of the entire gene sequence, generating accurate and reliable sequences with maximum coverage and high-quality genomic sequence data. Among the 20 amino acids, tyrosine (Tyr/Y), tryptophan (Trp/W), phenylalanine (Phe/F), and histidine (His/H) are aromatic amino acids, while cysteine (Cys/C) and methionine (Met/M) are sulfur-containing amino acids. Cysteine, tyrosine, tryptophan, and histidine all possess special groups that can serve as direct hydrogen donors, providing hydrogen atoms to free radicals and thereby achieving radical scavenging effects. The sulfur atom in methionine can donate electrons to free radicals, thereby stabilizing them [51]. In addition, hydrophobic amino acids such as valine (Val/V), proline (Pro/P), leucine (Leu/L), glycine (Gly/G), alanine (Ala/A), lysine (Lys/K), and methionine (Met/M) have been confirmed to possess strong antioxidant activity. The presence of hydrophobic amino acids in peptide sequences increases the contact area at the water–oil interface, facilitating the combination with free radicals and inhibiting oxidative reactions [52,53]. Liang Fan [54] demonstrated through experiments that peptides derived from black algae protein hydrolysates with higher hydrophobic amino acid content exhibited higher DPPH radical scavenging rates, all above 95%, while their ABTS and hydroxyl radical scavenging rates also remained at high levels, confirming that peptides with such amino acid sequences are peptides with demonstrated antioxidant properties in the DPPH assay. The compositional analysis of the peptides identified in the G-1 fraction revealed a high prevalence of hydrophobic and aromatic amino acid residues. This observation is consistent with the structural features commonly associated with antioxidant peptides reported in the literature [52,53], where hydrophobic residues are thought to enhance peptide accessibility to radical species at the water-lipid interface, and aromatic residues can act as hydrogen donors. However, it is critical to interpret this finding with appropriate caution: compositional analysis alone is insufficient to confirm antioxidant activity for any individual peptide. While the abundance of these residue types within the G-1 peptide population may partially explain the fraction’s strong DPPH scavenging capacity (81.74%), the specific contribution of each peptide remains unknown. To establish definitive sequence–activity relationships and to confirm the antioxidant activity of individual peptides, future studies should involve peptide synthesis, purification, and systematic in vitro activity evaluation. Existing studies have confirmed that quinoa peptides rich in hydrophobic and aromatic amino acids can maintain good antioxidant activity even after simulated gastrointestinal digestion in vitro, suggesting that these peptides may warrant further evaluation in in vivo models [55].

Since de novo sequencing was employed, a total of 3393 amino acid sequences with a confidence level above 80% and 1549 sequences with a confidence level above 90% were obtained. Although all peptide sequences contained amino acids with antioxidant activity, the large number of sequences necessitated the selection of a subset of peptides for further analysis based on activity requirements.

It should be noted that the present study did not involve the synthesis or functional validation of individual peptides, nor did it compare the frequency of hydrophobic residues with the parent quinoa protein sequence or a background dataset. Additionally, the predominance of peptides below 1000 Da may partly reflect an analytical bias inherent to de novo sequencing. Furthermore, the mass balance (extraction yield and protein content) was not systematically recorded during protein extraction, so the overall efficiency cannot be precisely reported. Nevertheless, the extraction conditions are consistent with established protocols [11], and subsequent experiments were performed on soluble hydrolysate fractions, which are independent of the absolute extraction yield. These limitations should be considered when interpreting the present data. Future work incorporating peptide synthesis, activity verification, and rigorous mass balance calculations is needed to validate the structure-activity relationships and techno-economic feasibility of the process.

## 5. Conclusions

This study investigated the preparation and sequence identification of quinoa antioxidant peptides, and the following conclusions were drawn. Among the three quinoa varieties, black quinoa was selected as the optimal raw material. In the enzymatic hydrolysis experiments, alcalase was identified as the best protease. Using response surface methodology, the optimal conditions for the preparation of antioxidant peptides were determined as follows: enzyme concentration 4000 U·g^−1^, hydrolysis time 3.5 h, temperature 42 °C, and pH 7.6. Under these conditions, the DPPH radical scavenging rate of the quinoa antioxidant peptides reached an average value of 86.61%.

After purification by Sephadex G-15 gel filtration chromatography, three elution peaks (G-1, G-2, G-3) were obtained. Among these, G-1 exhibited the highest antioxidant activity, with a DPPH radical scavenging rate of (81.74 ± 2.24)%.

The peptide fractions were analyzed by liquid chromatography–tandem mass spectrometry (LC-MS/MS), and the amino acid sequences were determined using de novo sequencing. The obtained fragments exhibited good radical-scavenging activity in the in vitro DPPH assay, suggesting the presence of antioxidant capacity within the G-1 peptide mixture. However, the specific contribution of individual peptides to this activity and their potential physiological relevance remain to be investigated through further cell-based and in vivo studies.

Using quinoa as the raw material, this study elucidated the optimal preparation process and activity-related sequence characteristics of quinoa antioxidant peptides, serving as a reference for quinoa product development. Further studies including in vivo validation and process economy analysis are needed to support industrial applications. Furthermore, previous studies have demonstrated that quinoa protein hydrolysates can exhibit antihypertensive and hypoglycemic activities. For instance, quinoa-derived peptides have been reported to possess ACE-inhibitory and antidiabetic potential [9], while other peptide fractions derived from quinoa protein have shown direct antihypertensive activity in vitro [56]. Given that some of the peptide sequences identified in the present study (e.g., those rich in hydrophobic and aromatic residues such as VVGF, LLGPAL, and LVGLF) share structural features with these previously reported multifunctional quinoa peptides, it is plausible that the quinoa antioxidant peptides obtained herein may possess additional bioactivities beyond antioxidant effects. However, this hypothesis requires dedicated experimental validation through targeted enzyme inhibition assays and in vivo studies, which are beyond the scope of the present work.

## Figures and Tables

**Table 1:** Optimum reaction conditions of various enzymes.

Type	Temperature (°C)	pH
Bromelain	55	7.0
papain	50	7.0
Flavourzyme	50	7.0
complex protease	50	6.0
alcalase	45	8.5
neutral protease	50	7.5
acid protease	40	2.5
trypsin	37	7.5

**Table 2:** Response surface test design table.

Level	Factors			
	A (U·g^−1^)	B (h)	C (°C)	D
1	−1	−1	−1	−1
2	0	0	0	0
3	1	1	1	1

**Table 3:** Experimental results of response surface.

Sequence Number	Enzyme Concentration (U·g^−1^)	Time (h)	Temperature (°C)	pH	Antioxidant Activity (%)
1	−1	−1	0	0	77.74 ± 1.07
2	1	−1	0	0	79.02 ± 0.89
3	−1	1	0	0	75.90 ± 0.68
4	1	1	0	0	76.90 ± 0.59
5	0	0	−1	−1	64.14 ± 0.99
6	0	0	1	−1	79.12 ± 1.13
7	0	0	−1	1	70.75 ± 1.62
8	0	0	1	1	51.88 ± 0.55
9	−1	0	0	−1	69.33 ± 0.93
10	1	0	0	−1	64.01 ± 0.67
11	−1	0	0	1	53.21 ± 2.28
12	1	0	0	1	60.99 ± 0.32
13	0	−1	−1	0	82.71 ± 0.68
14	0	0	−1	1	83.50 ± 1.10
15	0	−1	1	0	78.89 ± 0.77
16	0	1	1	0	81.14 ± 0.65
17	−1	0	−1	0	80.59 ± 0.39
18	1	0	−1	0	82.05 ± 0.94
19	−1	0	1	0	66.93 ± 0.88
20	1	0	1	0	78.25 ± 0.63
21	0	−1	0	−1	69.42 ± 1.01
22	0	1	0	−1	70.95 ± 2.06
23	0	−1	0	1	62.00 ± 0.80
24	0	1	0	1	65.90 ± 0.98
25	0	0	0	0	84.34 ± 1.02
26	0	0	0	0	86.79 ± 0.78
27	0	0	0	0	83.29 ± 0.82
28	0	0	0	0	85.63 ± 0.29
29	0	0	0	0	85.35 ± 0.91

**Table 4:** Variance and significance analysis.

Source	Sum of Squares	df	Mean Square	F-Value	*p*-Value	Significance
Model	2601.67	14	185.83	35.54	<0.0001	**
A	25.58	1	25.58	4.89	0.0441	*
B	1.70	1	1.70	0.32	0.5781	
C	63.16	1	63.16	12.08	0.0037	**
D	227.42	1	227.42	43.49	<0.0001	**
AB	0.020	1	0.020	3.748 × 10^−3^	0.9520	
AC	24.30	1	24.30	4.65	0.0490	*
AD	42.90	1	42.90	8.20	0.0125	*
BC	0.53	1	0.53	0.10	0.7543	
BD	1.40	1	1.40	0.27	0.6124	
CD	286.46	1	286.46	54.78	<0.0001	**
A^2^	258.54	1	258.54	49.44	<0.0001	**
B^2^	13.07	1	13.07	2.50	0.1362	
C^2^	24.28	1	24.28	4.64	0.0491	*
D^2^	1812.45	1	1812.45	346.61	<0.0001	**
Residual	73.21	14	5.23			
Lack of Fit	66.15	10	6.62	3.75	0.1071	
Pure Error	7.05	4	1.76			
Total	2674.88	28				

** indicates extremely significant difference (*p* < 0.01); * indicates significant difference (*p* < 0.05). R^2^ = 0.9726, Adj R^2^ = 0.9453. Explanation: enzyme concentration (A), time (B), temperature (C), and pH (D).

**Table 5:** Amino acid sequence.

Confidence Level	1*	2*	3*	4*	5*	Number of Peptides
99%	0	4	38	45	1	88
98%	0	10	54	100	5	169
97%	0	9	44	83	5	141
96	0	10	51	88	2	151
95	2	13	51	117	3	186
94	0	7	48	93	2	150
93	0	7	44	102	4	157
92	0	10	58	97	1	166
91	0	8	43	118	4	173
90	0	9	60	96	3	168
Number of peptides	2	87	491	9398	30	1549

1*: Peptide sequences containing one hydrophobic amino acid; 2*: Peptide sequences consisting entirely of hydrophobic amino acids; 3*: Peptide sequences containing both hydrophobic and aromatic amino acids; 4*: Peptide sequences containing two or more hydrophobic amino acids; 5*: Peptide sequences consisting entirely of hydrophobic and aromatic amino acids.

**Table 6:** Identified peptides in component G-1.

Sequence Number	Peptide Sequence	Confidence (%)	Length (aa)	*m*/*z*	Molecular Weight (Da)	Error (ppm)
1	VVGF	99	4	421.2439	420.2372	−1.5
2	LLGPAL	99	6	583.3809	582.3741	−0.9
3	MPAGVSHW	99	8	868.4131	867.4061	−0.4
4	LLPH	98	4	479.2978	478.2903	0.4
5	LAVGL	98	5	472.3133	471.3057	0.8
6	ASVGFKA	98	7	679.3757	678.3701	−2.4
7	WVAL	97	4	488.287	487.2794	0.7
8	PAGVAH	97	6	551.293	550.2863	−1.1
9	LVGLF	96	5	548.3455	547.337	2.2
10	KMTTVH	96	6	758.3845	757.3793	−2.7
11	FDFHEK	95	6	822.3779	821.3708	−0.2
12	LVVLGH	95	6	319.2051	636.3959	−0.4
13	FMHGKLE	95	7	431.2175	860.4214	−1.1
14	FMAM	94	4	499.2042	498.1971	−0.4
15	YLPLL	94	5	618.387	617.3788	1.4
16	VLPGVVGF	93	8	787.4701	786.4639	−1.4
17	LVAF	93	4	449.2762	448.2686	0.7
18	LLKAL	92	5	279.2038	556.3948	−3.1
19	MFGAP	92	5	522.2383	521.2308	0.6
20	FAYPGLL	91	7	780.4282	779.4218	−1.0

## Data Availability

The original contributions presented in this study are included in the article/Supplementary Materials. Further inquiries can be directed to the corresponding authors.

## Supplementary Materials

The following supporting information can be downloaded at: https://www.mdpi.com/article/10.3390/foods15173124/s1, Figure S1: Total ion chromatogram (TIC) of the G-1 fraction obtained by LC-MS/MS analysis.
