# Supplementary material for: Preparation and Identification of Antioxidant Peptides in Quinoa
Source: Foods. 2026 Sep 2;15(17):3124. doi: 10.3390/foods15173124 (PMC13565606; doi:10.3390/foods15173124)
Supplement: Supplementary file 1 [file foods-15-03124-s001.zip › foods-4456647-supplementary.pdf]

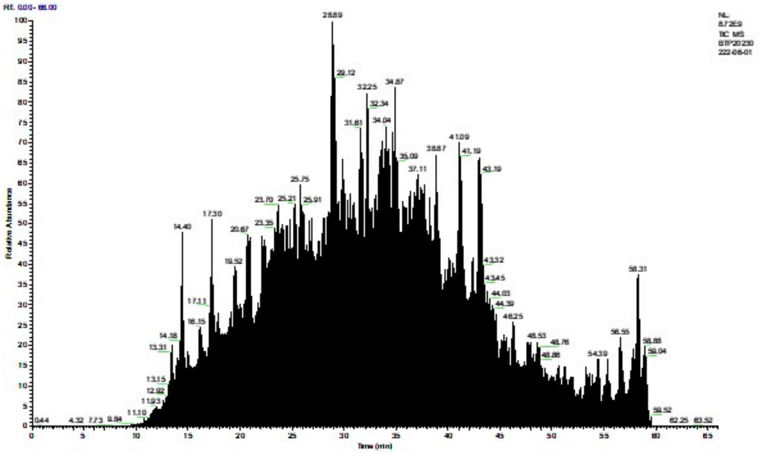

Figure S1. Total ion chromatogram (TIC) of the G-1 fraction obtained by LC-MS/MS analysis.
